# Association between atherogenic index of plasma and physical dysfunction: a cross-sectional study of middle-aged and older adults in China

**DOI:** 10.3389/fpubh.2025.1580340

**Published:** 2025-05-30

**Authors:** Jianghui Zhou, Yun Chang, Hechen Shen, Meng Zhang, Yuchao Wang, Xiaoyu Liang, Wenqing Gao

**Affiliations:** ^1^The Third Central Clinical College of Tianjin Medical University, Tianjin, China; ^2^Department of Heart Center, The Third Central Hospital of Tianjin, Tianjin, China; ^3^Tianjin Key Laboratory of Extracorporeal Life Support for Critical Diseases, Tianjin, China; ^4^Tianjin ECMO Treatment and Training Base, Tianjin, China; ^5^Artificial Cell Engineering Technology Research Center, Tianjin, China; ^6^School of Medicine, Nankai University, Tianjin, China

**Keywords:** atherogenic index of plasma, physical dysfunction, restricted cubic spline regression, cross-sectional study, older adult population, lipid metabolism

## Abstract

**Background:**

Physical dysfunction is common in older adults and increases disease risk. The atherogenic index of plasma (AIP) is a promising biomarker for this condition. This study explored the dose–response relationship between AIP and physical dysfunction.

**Methods:**

Data from 11,369 CHARLS participants (aged ≥45 years) in 2015 and 2018 were analyzed using univariate and multivariate logistic regression, adjusting for demographics and lifestyle factors. The restricted cubic splines were used to examine possible non-linear associations and visualize the dose–response relationship between AIP and physical dysfunction. ROC curve analysis assessed AIP’s predictive performance, and subgroup analyses evaluated interactions.

**Results:**

Each interquartile range (IQR) increase in AIP was associated with a 13.4% higher odds of physical dysfunction (adjusted OR = 1.134, 95% CI: 1.066–1.207, *p* < 0.001), with a dose–response threshold identified at an AIP value of approximately 0.37. Beyond this threshold, the odds of physical dysfunction increased steadily, confirming a non-linear relationship. AIP exhibited moderate predictive accuracy for physical dysfunction (AUC = 0.748, 95% CI: 0.738–0.758). Stratified analysis showed AIP was significantly linked to higher physical dysfunction risk in subgroups including those aged <65, females, married individuals, high school or college-educated, rural residents, non-smokers, and non-drinkers (*p* < 0.05), with no significance in other subgroups. Interaction analysis identified marital status (*p* = 0.035) and education level (*p* = 0.034) as significant effect modifiers, where subgroup differences notably altered the AIP-dysfunction association, warranting further study.

**Conclusion:**

Elevated AIP is significantly associated with increased physical dysfunction risk, highlighting its potential as a simple, predictive biomarker.

## 1 Introduction

With the global population aging at an unprecedented rate, physical dysfunction has become a pressing public health concern, affecting millions of older adults worldwide. Based on China’s national conditions and the current situation of the middle-aged and older adult population, we generally define those aged 45–64 as middle-aged and those aged 65 and above as older adults (1). By 2021, the scale of older adults with physical disability in China had reached 45.3 million, with a disability rate of 17%, and the total annual cost was 1.35 trillion yuan, accounting for about 1% of the national GDP in that year (according to the release of the China Elderly Health Report (2024) sponsored by the National Institute of Health and Medical Big Data of Wuhan University & National Bureau of Statistics of China, 2023).^1^ And it is projected that the number of older people with tertiary disability requiring complex care may increase by 39%, from 45.3 million in 2020 to 59.32 million in 2030 (2). This condition is characterized by a decline in the ability to perform essential daily activities, such as mobility, self-care, and household tasks, leading to reduced independence and a lower quality of life (3, 4). Moreover, physical dysfunction is strongly associated with the onset and progression of chronic diseases, including cardiovascular disease, diabetes, and metabolic syndrome (5). These health complications not only increase morbidity and mortality but also place a significant burden on healthcare systems and caregivers. As physiological and metabolic changes accelerate with aging, older individuals experience progressive declines in muscle strength, joint flexibility, and balance, which heighten their vulnerability to functional disorders, especially when exposed to external stressors or health challenges (3, 6). The cumulative impact of these impairments can lead to social isolation, loss of autonomy, and deteriorating mental health, further exacerbating overall well-being. Given the profound implications of physical dysfunction, early identification of modifiable risk factors is critical for implementing preventive strategies. Timely intervention can delay functional decline, improve health outcomes, and enable targeted approaches to reduce the burden of chronic diseases (7).

Recent research on physical dysfunction in older adults has increasingly focused on identifying reliable biomarkers and predictive factors. Commonly used indicators include measures of muscle strength, body composition, and inflammatory markers, which are associated with the risk of chronic disease and functional decline (8). However, there is growing interest in lipid metabolism-related biomarkers, given their well-documented role in chronic diseases such as cardiovascular and metabolic disorders (9, 10). The atherogenic index of plasma (AIP), defined as the logarithm of the ratio of triglycerides to high-density lipoprotein cholesterol (TG/HDL-C), has emerged as a key marker of lipid metabolism abnormalities (9). AIP has been widely recognized for its strong association with cardiovascular risk, metabolic syndrome, and atherosclerosis (10). Elevated AIP levels are linked to endothelial dysfunction, systemic inflammation, and insulin resistance, all of which contribute to the progression of chronic diseases (10). In light of these findings, researchers have begun to hypothesize that AIP may also play a role in physical dysfunction. Emerging studies suggest that increased AIP levels are correlated with functional impairments such as reduced muscle strength, poor physical performance, and unstable gait in older adults (8). Despite these preliminary findings, the direct evidence establishing AIP as a risk factor for physical dysfunction remains limited and inconclusive. Given the importance of early detection and prevention of functional decline, further research is needed to clarify the relationship between AIP and physical dysfunction. Investigating this association may provide a new perspective on the pathophysiology of functional disorders and offer a novel, easily measurable biomarker for risk assessment and personalized interventions in aging populations (9). Emerging cohort studies have linked the Atherogenic Index of Plasma (AIP) to muscle strength decline and sarcopenia (11), yet no nationally representative research has examined its predictive value for multidimensional physical dysfunction in older adults.

This study aims to investigate the relationship between the atherogenic index of plasma (AIP) and physical dysfunction in middle-aged and older adult populations. Using a cross-sectional design, we analyze data from the China Health and Retirement Longitudinal Study (CHARLS), which includes a large, nationally representative sample of individuals aged 45 years and older. The study evaluates key variables such as AIP levels, measures of physical function, and potential confounding factors, including demographic characteristics, lifestyle behaviors, and chronic disease history. Our objective is to determine whether AIP can serve as a predictive biomarker for physical dysfunction, filling a critical gap in current research. By identifying associations between AIP and functional decline, this study seeks to provide evidence that supports the early detection of high-risk individuals.

## 2 Methods

### 2.1 Study design and population

This study utilized a cross-sectional design based on data from the 2015 and 2018 waves of the China Health and Retirement Longitudinal Study (CHARLS) (12).^2^ A total of 11,369 participants aged 45 years and older were included. The sample represented a diverse demographic, encompassing various age groups, genders, educational backgrounds, and lifestyle factors such as smoking and alcohol consumption. At the time of the CHARLS survey, written informed consent was obtained from all respondents, and the original study was approved by the ethics committee. As this analysis constitutes secondary data analysis, no additional ethics approval was required.

### 2.2 Atherogenic index of plasma

The atherogenic index of plasma (AIP) was calculated using the formula:

$\text{AIP}=\log10(\frac{\mathrm{TG}}{\mathrm{HDL}-C})$.

where TG denotes serum triglycerides and HDL-C represents high-density lipoprotein cholesterol, both measured in consistent units (mmol/L or mg/dL). AIP was used to assess lipid metabolism and cardiovascular risk, with participants stratified into four quartiles (Q1–Q4) to evaluate potential dose–response effects. AIP values were divided into quartiles (Q1–Q4) by sorting the data in ascending order and equally partitioning the study population: Q1 represents the 25th percentile (lower quartile), Q2 the 50th percentile (median), Q3 the 75th percentile (upper quartile), and Q4 the highest 25% of values. The interquartile range (IQR) of AIP was calculated as Q3 minus Q1 (IQR = Q3 − Q1). The ratio of AIP to its IQR (AIP/IQR) was used to measure the dispersion of individual data points relative to the central 50% of the distribution, indicating how far each value lies from the median in terms of IQR multiples. Clinically, AIP is categorized into three risk levels according to Chinese guidelines: values below 0.11 indicate low risk, between 0.11 and 0.21 indicate moderate risk, and above 0.21 indicate high risk for atherosclerosis (13). The formula incorporates a logarithmic transformation to mitigate the effects of skewed distributions in the TG/HDL-C ratio. AIP has demonstrated significant predictive value in cardiovascular health, with studies showing that a 0.1-unit increase in AIP correlates with a 14% higher risk of cardiovascular events, according to the Chinese Guidelines for the Prevention and Treatment of Dyslipidemia in Adults (14).

### 2.3 Physical dysfunction

The definition of physical dysfunction in the CHARLS questionnaire is structured around 9 physical-function-related activities. These encompass actions like running or jogging for 1 km, walking 1 km or 100 meters, rising from a chair after extended sitting, climbing several floors consecutively, bending over, bending the knees or squatting, stretching arms upward along the shoulders, and picking up a small coin from a table.

Each activity-related question offers four response choices: (1) No difficulty; (2) Difficulty encountered but still achievable; (3) Difficulty present requiring assistance; (4) Incapable of completion. A participant is classified as having physical dysfunction if they report difficulty with any one of these nine activities (15).

Participants were categorized into two groups: Physical dysfunction present (1) and No physical dysfunction (0).

### 2.4 Covariates

Covariates included predisposing factors (age, sex), enabling factors (education, residential location), and health need-related factors (chronic conditions), systematically categorizing demographic, socioeconomic, and health status determinants to explore their collective influence on the association. Additionally, other elements such as demographic factors (marital status), health behaviors (smoking, alcohol consumption), and healthcare access (insurance status, distance to nearest clinic) were incorporated. In multivariate regression analyses, these covariates were adjusted for to control for potential confounding effects. This comprehensive approach ensured more accurate results by accounting for a wide range of influences, including age, sex, education level, marital status, residence, smoking, alcohol consumption, and chronic conditions.

### 2.5 Statistical analysis

#### 2.5.1 Data preprocessing

This study utilized baseline data from the China Health and Retirement Longitudinal Study (CHARLS), a nationally representative survey of Chinese residents, which employed a multistage probability proportional to size (PPS) sampling design to select participants from 28 provinces (12).

Data preprocessing involved three sequential steps to ensure analytical validity. First, missing values in key variables—atherogenic index of plasma (AIP), physical dysfunction assessments, and core covariates (age, sex, education)—were handled via listwise deletion, retaining 11,369 complete cases. Second, AIP was log₁₀-transformed (AIP = log₁₀(TG/HDL-C)) to normalize its skewed distribution, while continuous variables like age were kept in original units to maintain clinical interpretability.

#### 2.5.2 Logistic regression models

We employed a hierarchical logistic regression framework to systematically evaluate covariate-adjusted associations. Three nested models were constructed: Model 1 (unadjusted), Model 2 (adjusted for demographics), Model 3 (full adjustment including behaviors and comorbidities).

**Table:** 

Model	Variables included	Objective
Model 1	AIP (continuous variable, per IQR increment)	Crude association
Model 2	Model 1 + Demographics (age, sex, education level, marital status, urban/rural residence)	Control socio-demographic confounding
Model 3	Model 2 + Health behaviors (smoking status [current/former/never], alcohol frequency [≥1 drink/month]) + Number of chronic diseases (0/1/≥2)	Fully adjusted model (all confounders within theoretical framework)

The number of chronic conditions was defined as the count of the following chronic diseases that an individual had (hypertension, diabetes, heart disease, stroke, arthritis, asthma, etc.), and categorized into 0, 1, or ≥2 conditions. The odds ratios (ORs) and 95% confidence intervals (CIs) were reported for both continuous (per IQR increase) and categorical (quartiles) AIP variables.

#### 2.5.3 Nonlinear relation test

Restricted cubic spline (RCS) analysis was applied to model the non-linear dose–response relationship between AIP and physical dysfunction, identifying critical thresholds where risk increases significantly. An RCS plot visualized the continuous association, with the x-axis representing AIP, the y-axis showing odds ratios (ORs) of dysfunction, and shaded 95% confidence intervals (CIs) reflecting uncertainty. Nonlinearity was confirmed via a likelihood ratio test (*p* < 0.05), after which the inflection point of AIP—marking the optimal threshold for risk stratification—was extracted.

#### 2.5.4 Validation of predictive efficacy

The predictive ability of AIP for identifying physical dysfunction was evaluated through Receiver Operating Characteristic (ROC) curve analysis, which assessed the sensitivity (true-positive rate) and specificity (true-negative rate) of AIP in distinguishing individuals with vs. without dysfunction. AUCs for AIP alone and combined covariates were calculated using the predicted probabilities of Model 3. The area under the curve (AUC) was used to measure model performance, with values closer to 1 indicating greater discriminative power.

#### 2.5.5 Stratified analysis

The stratification criteria, including gender, age, educational attainment, marital status, residential location (urban/rural), drinking status and smoking status, were determined through theoretical considerations and univariate analyses.

Analytical procedures comprised the following sequential steps: First, the study population was stratified according to the predefined demographic variables. Subsequently, Model 3 (the fully adjusted multivariate logistic regression model) was independently fitted to each subgroup. This stratified analysis enabled calculation of adjusted odds ratios (ORs) with corresponding 95% confidence intervals (CIs) for each demographic stratum (shown by forest plot).

#### 2.5.6 Interaction analysis

Interaction effects between AIP and key covariates were examined to assess potential effect modification. This was implemented by incorporating multiplicative interaction terms (“AIP × stratifying variable”) into the fully adjusted Model 3. Statistical significance of interactions was evaluated through likelihood ratio tests comparing models with versus without interaction terms, using a significance threshold of *p* < 0.05. All interaction analyses maintained the scheme and covariate adjustment structure of the Model 3.

#### 2.5.7 Statistical methods: software, tests, and significance

Continuous variables were tested for normality using the Kolmogorov–Smirnov test. Variables following a normal distribution were described as mean ± standard deviation, with between-group comparisons performed using t-tests. Skewed continuous variables were reported as median (interquartile range, IQR), and between-group differences were assessed using the Mann–Whitney U test. Categorical variables were summarized as frequencies and percentages, with between-group comparisons conducted via chi-squared tests or Fisher’s exact tests as appropriate. All statistical analyses were performed using SPSS (version 29.0) and R (version 4.3.1). Statistical significance was defined as a *p* value < 0.05.

## 3 Results

### 3.1 Baseline characteristics

Table 1 presents the baseline characteristics of 11,369 participants, categorized by the presence or absence of physical dysfunction. Among them, 3,042 participants (26.77%) were in the non-physical dysfunction group, while 8,327 participants (73.23%) were in the physical dysfunction group.

**Table 1:** Baseline characteristics of participants categorized by the presence or absence of physical dysfunction (*N* = 11,369).

Stratified by physical dysfunction					
	Level	Overall	Non-physical dysfunction	Physical dysfunction	*p*-value
n		11369	3042	8327	
chronic [mean (SD)]		1.245 (1.280)	0.693 (0.908)	1.446 (1.336)	<0.001
Number of chronic conditions (%)	≥2	3971 (34.928)	515 (16.930)	3456 (41.504)	<0.001
	0	4020 (35.359)	1633 (53.682)	2387 (28.666)	
	1	3378 (29.712)	894 (29.389)	2484 (29.831)	
Sex (%)	Female	6085 (53.523)	1157 (38.034)	4928 (59.181)	<0.001
	Male	5284 (46.477)	1885 (61.966)	3399 (40.819)	
Physical Dysfunction [mean (SD)]		0.732 (0.443)	0.000 (0.000)	1.000 (0.000)	<0.001
Marital (%)	Married	9915 (87.211)	2813 (92.472)	7102 (85.289)	<0.001
	Non-married	1454 (12.789)	229 (7.528)	1225 (14.711)	
Education (%)	College or above	348 (3.061)	136 (4.471)	212 (2.546)	<0.001
	High school	3002 (26.405)	1092 (35.897)	1910 (22.937)	
	Primary school or below	8019 (70.534)	1814 (59.632)	6205 (74.517)	
Location (%)	City/town	4211 (37.039)	1231 (40.467)	2980 (35.787)	<0.001
	Village	7158 (62.961)	1811 (59.533)	5347 (64.213)	
Smoking (%)	Current smoker	3136 (27.584)	1093 (35.930)	2043 (24.535)	<0.001
	Ex-smoker	1743 (15.331)	459 (15.089)	1284 (15.420)	
	Non-smoker	6490 (57.085)	1490 (48.981)	5000 (60.046)	
Drinking (%)	Drink but less than once a month	991 (8.717)	314 (10.322)	677 (8.130)	<0.001
	Drink more than once a month	2989 (26.291)	1127 (37.048)	1862 (22.361)	
	None of these	7389 (64.993)	1601 (52.630)	5788 (69.509)	
Age [mean (SD)]		60.266 (9.444)	56.608 (8.144)	61.602 (9.533)	<0.001
BMI [mean (SD)]		24.582 (15.887)	24.136 (8.592)	24.745 (17.827)	0.073
AIP [mean (SD)]		0.390 (0.282)	0.372 (0.283)	0.396 (0.282)	<0.001
AIP_Q (%)	Q1	2846 (25.033)	812 (26.693)	2034 (24.427)	0.001
	Q2	2839 (24.971)	794 (26.101)	2045 (24.559)	
	Q3	2842 (24.998)	752 (24.721)	2090 (25.099)	
	Q4	2842 (24.998)	684 (22.485)	2158 (25.916)	
AIP per IQR [mean (SD)]		1.022 (0.740)	0.976 (0.742)	1.039 (0.739)	<0.001

Data are presented as counts and percentages for categorical variables and means with standard deviations for continuous variables. Statistically significant differences between the physical dysfunction and non-dysfunction groups were observed for age, sex, education level, marital status, residence, smoking status, drinking frequency, number of chronic conditions and AIP quartile distribution (*p* < 0.001 for all comparisons). BMI showed no significant difference (*p* = 0.073). All quartiles of AIP were determined based on the distribution of AIP values among all participants. The quartiles of AIP are divided at the nodes 0.1666, 0.3397, and 0.5335. Specifically, Q1 represents values below 0.1666, Q2 ranges from 0.1666 to 0.3397, Q3 from 0.3397 to 0.5335, and Q4 consists of values above 0.5335.

Participants with physical dysfunction were significantly older, with a mean age of 61.60 years compared to 56.61 years in the non-dysfunction group (*p* < 0.001). Females made up a higher proportion of the physical dysfunction group (59.18%) compared to the non-dysfunction group (38.03%; *p* < 0.001). Educational attainment also differed notably between groups, with only 2.55% of participants in the physical dysfunction group having a college degree or higher, compared to 4.47% in the non-dysfunction group (*p* < 0.001). Additionally, individuals in the physical dysfunction group were more likely to live in rural areas (64.21% vs. 59.53%, *p* < 0.001) and were less likely to be married (85.29% vs. 92.47%, *p* < 0.001).

Regarding health and lifestyle factors, participants with physical dysfunction had a higher prevalence of chronic conditions. The proportion with ≥2 chronic conditions was 41.50% in the physical dysfunction group versus 16.93% in the non-dysfunction group (*p* < 0.001). They were less likely to be current smokers (24.54% vs. 35.93%, *p* < 0.001) and less likely to drink alcohol more than once a month (22.36% vs. 37.05%, *p* < 0.001).

The mean AIP value was 0.372 in the non-dysfunction group and 0.396 in the physical dysfunction group (*p* < 0.001). For AIP quartile distribution, the physical dysfunction group had a higher proportion in the highest quartile (25.92% vs. 22.49%, *p* = 0.001). The AIP_per_IQR value was 1.04 in the physical dysfunction group, higher than 0.98 in the non-dysfunction group (*p* < 0.001). No significant difference was observed in BMI between the two groups (*p* = 0.073).

These results indicate clear differences in demographic characteristics, health conditions, and lifestyle behaviors between those with and without physical dysfunction. Although BMI showed no significant difference, the higher chronic disease burden and distinct AIP-related metrics in the physical dysfunction group suggest a potential link between elevated AIP and greater risk of functional impairment.

### 3.2 Association between AIP and physical dysfunction

The association between AIP and physical dysfunction was examined using logistic regression analysis (Table 2).

**Table 2:** Logistic regression analysis: association between AIP and physical dysfunction.

Variable	Model 1	Model 2	Model 3	P for trend
AIP per IQR	1.123 (1.061–1.189)***	1.221 (1.150–1.297)***	1.134 (1.066–1.207)***	–
Q2 vs. Q1	1.028 (0.916–1.154)	1.040 (0.920–1.176)	1.003 (0.885–1.137)	
Q3 vs. Q1	1.110 (0.988–1.247)	1.141 (1.009–1.292)*	1.036 (0.912–1.176)	
Q4 vs. Q1	1.260 (1.119–1.418)***	1.475 (1.300–1.675)***	1.279 (1.123–1.458)***	
p for trend	1.079 (1.040–1.120)***	1.132 (1.088–1.178)***	1.078 (1.035–1.124)***	<0.001

Logistic regression analysis of the association between AIP and physical dysfunction. This table presents the odds ratios (ORs) and 95% confidence intervals (CIs) for the relationship between AIP and physical dysfunction across three models. ***p < 0.001, **p < 0.01, *p < 0.05. AIP is analyzed both as a continuous variable (per IQR increase) and as a categorical variable (quartiles). The dose–response trend is statistically significant (P for trend < 0.001).

**Model 1 (Unadjusted):**

In the unadjusted model including only AIP (continuous, per interquartile range [IQR] increase), each IQR elevation in AIP was significantly associated with a 12.3% higher odds of physical dysfunction (OR = 1.123, 95% CI: 1.061–1.189, *p* < 0.001), confirming the existence of a crude association.

**Model 2 (Demographically Adjusted):**

After adjusting for demographic factors (age, sex, education level, marital status, and urban/rural residence), the association strengthened (OR = 1.221, 95% CI: 1.150–1.297, *p* < 0.001), suggesting that demographic characteristics (e.g., older age, female sex, lower education) acted as confounders in the AIP-dysfunction relationship.

**Model 3 (Fully Adjusted):**

Further adjustment for health behaviors (smoking, alcohol consumption) and the number of chronic conditions (0, 1, ≥2) revealed a robust association between AIP and physical dysfunction (OR = 1.134, 95% CI: 1.066–1.207, *p* < 0.001), even after accounting for potential mediators (e.g., chronic diseases). This finding indicates that AIP independently predicts physical dysfunction beyond lifestyle and disease status.

Quartile analysis of AIP revealed a significant linear trend (*p* < 0.001). Compared with the lowest quartile (Q1, below 0.1666), the highest quartile (Q4, above 0.5335) in Model 3 showed a 27.9% increased risk of physical dysfunction (OR = 1.279, 95% CI: 1.123–1.458, *p* < 0.001). Notably, across models, the effect for Q4 vs. Q1 strengthens: Model 1 (OR = 1.260, 1.119–1.418, *p* < 0.001) → Model 3 (OR = 1.279, 1.123–1.458, *p* < 0.001). These results suggest the cumulative impact of elevated AIP on the likelihood of physical dysfunction, emphasizing its potential role as an independent risk factor.

The fully adjusted regression analysis (Table 3) revealed several significant covariates associated with physical dysfunction risk. Each one-year increase in age was associated with a higher likelihood of physical dysfunction (OR = 1.053, 95% CI [1.047, 1.059], *p* < 0.001). Males were at significantly lower risk than females (OR = 0.369, 95% CI [0.315, 0.430], *p* < 0.001).

**Table 3:** Effects of key covariates on physical dysfunction (Fully adjusted Model 3).

Category	Variable	OR (95% CI)	*p*-value
Demographic Factors	Age (per year)	1.053 (1.047–1.059)***	<0.001
Demographic Factors	Male vs. Female	0.369 (0.315–0.430)***	<0.001
Education Level	High school vs. College	1.468 (1.119–1.921)**	0.005
Education Level	Primary or below vs. College	2.158 (1.651–2.814)***	<0.001
Demographic Factors	Village vs. City	1.096 (0.992–1.210)	0.072
Demographic Factors	Married vs. Non-married	0.858 (0.722–1.017)	0.08
Chronic Diseases	1 vs. 0 chronic conditions	1.292 (1.144–1.461)***	<0.001
Chronic Diseases	≥2 vs. 0 chronic conditions	3.024 (2.693–3.396)***	<0.001
Health Behaviors	Ex-smoker vs. Non-smoker	1.514 (1.268–1.810)***	<0.001
Health Behaviors	Current smoker vs. Non-smoker	1.313 (1.125–1.533)***	<0.001
Health Behaviors	Drink more than once a month vs. None of these	0.750 (0.669–0.841)***	<0.001

This table presents odds ratios (ORs) and 95% confidence intervals (CIs) for the influence of demographic factors, education level, health behaviors, and chronic diseases on physical dysfunction. The fully adjusted model accounts for AIP, age, sex, smoking, alcohol consumption, and other relevant covariates. Statistically significant associations are indicated by their *p*-values, with *p* < 0.05 denoting significance and *p* < 0.001 indicating stronger associations. ***p < 0.001, **p < 0.01.

Education level also played a significant role. Participants with primary school education or below had over double the risk compared to those with a college education (OR = 2.158, 95% CI [1.651, 2.814], *p* < 0.001), while those with a high-school education had a moderately increased risk (OR = 1.468, 95% CI [1.119, 1.921], *p* = 0.005). In terms of health behaviors, non-smokers showed a lower risk of physical dysfunction compared to current smokers (OR = 1.313, 95% CI [1.125, 1.533], *p* < 0.001 for current smokers vs. non-smokers), but ex-smokers demonstrated a higher risk (OR = 1.514, 95% CI [1.268, 1.810], *p* < 0.001 for ex-smokers vs. non-smokers). Additionally, those who drink more than once a month had a lower risk compared to those in the “none of these” category (OR = 0.750, 95% CI [0.669, 0.841], *p* < 0.001).

Chronic disease burden was strongly associated with dysfunction risk. Participants with one chronic condition had higher odds compared to those with none (OR = 1.292, 95% CI [1.144, 1.461], *p* < 0.001), and those with two or more chronic conditions had even higher odds (OR = 3.024, 95% CI [2.693, 3.396], *p* < 0.001). Participants living in rural areas had a non-significant higher odds trend compared to those in urban areas (OR = 1.096, 95% CI [0.992, 1.210], *p* = 0.072). Marital status showed a non-significant trend (OR = 0.858, 95% CI [0.722, 1.017], *p* = 0.08), indicating no strong association with physical dysfunction in this model.

### 3.3 Non-linear relationship between AIP and physical dysfunction

**Figure 1:**
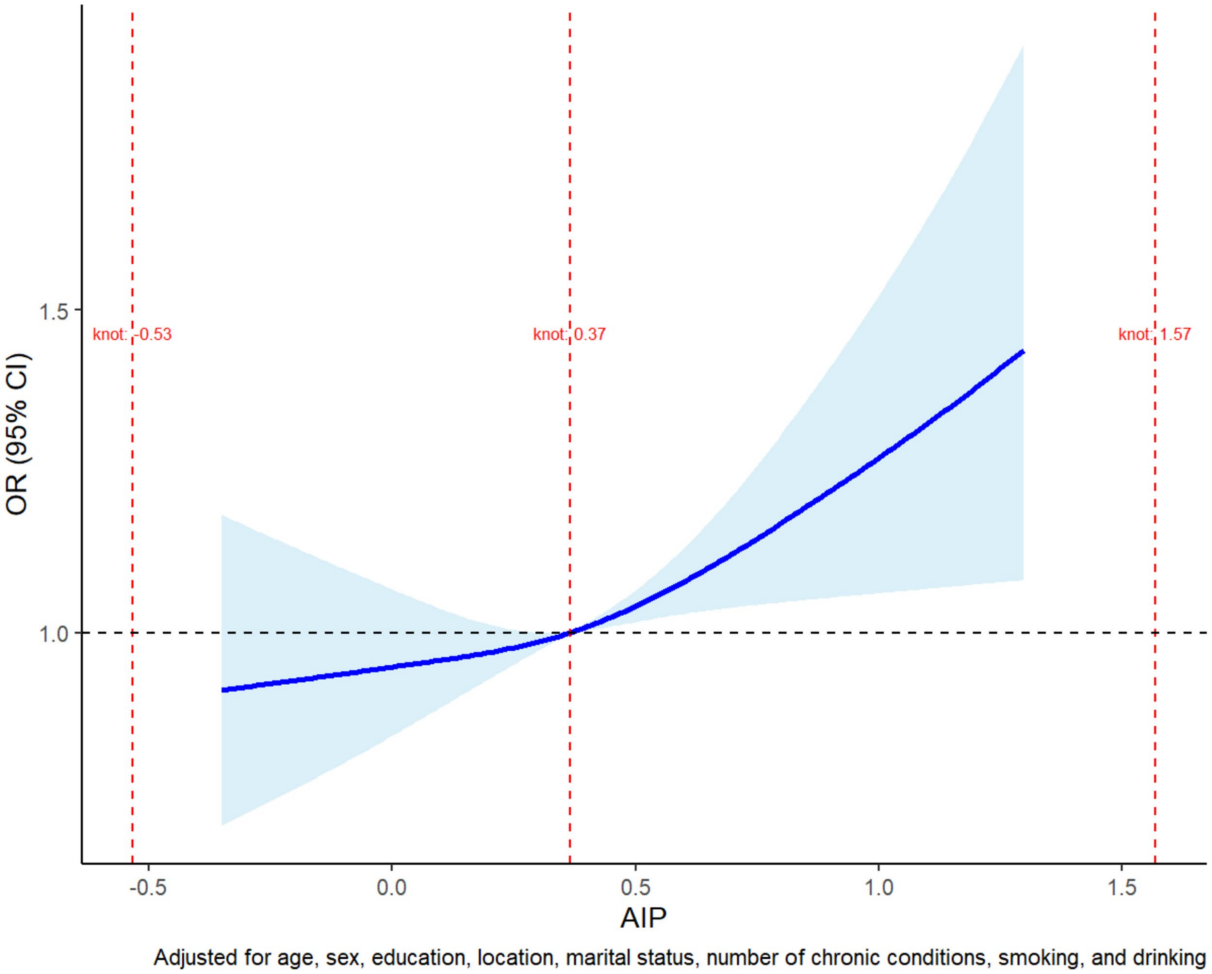
Restricted cubic spline plot of the non-linear association between AIP and physical dysfunction risk. The plot shows the adjusted odds ratio (OR) for physical dysfunction across AIP values. The risk remained stable at lower AIP levels but began to increase steadily after a threshold of approximately 0.37. The shaded region represents the 95% confidence interval, with wider intervals at higher AIP values due to limited data. The model was adjusted for age, sex, education, marital status, residence, smoking, alcohol consumption, and chronic conditions.

A restricted cubic spline regression model was employed to investigate the potential non-linear relationship between AIP and physical dysfunction risk (Figure 1). The analysis demonstrated that the odds ratio (OR) for physical dysfunction remained close to 1.0 at lower AIP values, indicating minimal risk. However, once AIP exceeded approximately 0.37, the OR increased steadily, suggesting a threshold effect where higher AIP values are associated with a progressively elevated risk of physical dysfunction. Beyond an AIP value of 0.5, the 95% confidence interval widened, reflecting reduced precision due to sparse data in this range. These findings highlight the importance of monitoring elevated AIP levels, as they may serve as an early indicator of functional decline, emphasizing the need for targeted interventions to mitigate health risks associated with lipid metabolism dysregulation.

### 3.4 ROC curve analysis

**Figure 2:**
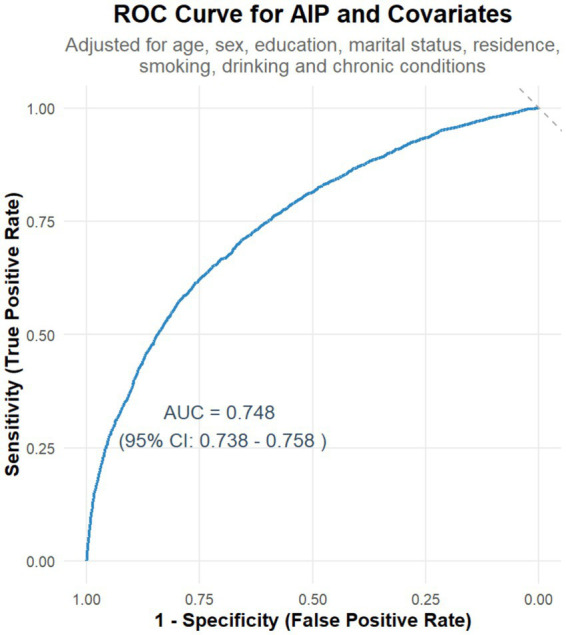
Receiver operating characteristic (ROC) curve for AIP and covariates in predicting physical dysfunction. The curve shows the sensitivity and specificity of AIP for predicting physical dysfunction. The area under the curve (AUC) is 0.748, indicating moderate predictive performance. The analysis was adjusted for relevant covariates, including age, sex, education, marital status, residence, smoking, drinking, and chronic conditions.

A receiver operating characteristic (ROC) curve was generated to evaluate the predictive ability of AIP for identifying physical dysfunction. In the unadjusted analysis, AIP alone yielded a modest area under the curve (AUC) of 0.525 (95% CI: 0.513–0.537), reflecting limited discriminative power when used in isolation (Supplementary Figure S1). Conversely, when incorporated into a multivariable model adjusted for key covariates (e.g., age, chronic conditions, socioeconomic factors), the AUC improved substantially to 0.748 (95% CI: 0.738–0.758; Figure 2), indicating moderate discriminative capacity for distinguishing individuals with vs. without physical dysfunction.

### 3.5 Stratified analysis and interaction analysis

A comprehensive forest plot was constructed to integrate the findings from stratified and interaction analyses, illustrating the heterogeneity in the association between AIP and physical dysfunction across different sociodemographic and behavioral subgroups. The forest plot highlights variations in the strength of the association between AIP and physical dysfunction across subgroups:

Stratified analysis was conducted to explore the association between AIP and physical dysfunction across various subgroups.

- For age, participants aged <65 demonstrated a significant positive association with physical dysfunction (OR = 1.11, 95% CI [1.04, 1.20], *p* < 0.05), whereas those aged ≥65 did not (OR = 0.98, 95% CI [0.84, 1.14], *p* > 0.05).
- In terms of gender, females showed a significant positive association (OR = 1.15, 95% CI [1.04, 1.27], *p* < 0.05), while males did not (OR = 1.08, 95% CI [0.99, 1.18], *p* > 0.05).
- Regarding marital status, married individuals had a significant positive association (OR = 1.13, 95% CI [1.06, 1.21], *p* < 0.05), but non-married individuals did not (OR = 0.90, 95% CI [0.71, 1.14], *p* > 0.05).
- For education level, both high-school (OR = 1.25, 95% CI [1.11, 1.40], *p* < 0.05) and college-above (OR = 1.61, 95% CI [1.11, 2.38], *p* < 0.05) groups showed significant positive associations, while the primary-school group did not (OR = 1.03, 95% CI [0.95, 1.12], *p* > 0.05).
- In terms of residence, village residents had a significant positive association (OR = 1.15, 95% CI [1.05, 1.25], *p* < 0.05), but city/town residents did not (OR = 1.06, 95% CI [0.96, 1.18], *p* > 0.05).
- Regarding smoking status, non-smokers showed a significant positive association (OR = 1.14, 95% CI [1.04, 1.25], *p* < 0.05), while ex-smokers (OR = 1.14, 95% CI [0.96, 1.35], *p* > 0.05) and current smokers (OR = 1.05, 95% CI [0.94, 1.17], *p* > 0.05) did not.
- For drinking status, non-drinkers had a significant positive association (OR = 1.12, 95% CI [1.03, 1.22], *p* < 0.05), whereas drinkers did not (OR = 1.08, 95% CI [0.97, 1.19], *p* > 0.05).

These results indicate that AIP is significantly associated with physical dysfunction in subgroups such as younger individuals (Age < 65), females, married individuals, those with higher education (High school and College or above), village residents, non-smokers, and non-drinkers.

Interaction analysis was conducted to determine whether variables significantly modified the association between AIP and physical dysfunction. The results revealed that Marital Status had a *p*-value of 0.035, demonstrating a significant interaction, which indicated that it was an effect-modifying factor. Specifically, the association between AIP and physical dysfunction varied significantly across different marital status subgroups. Similarly, Education Level had a *p*-value of 0.034, showing a significant interaction as well. This meant that Education Level significantly altered the impact of AIP on physical dysfunction, with notable differences in the association across its various subgroups. These findings highlight that both Marital Status and Education Level are effect modifiers, and their different subgroups significantly changed the association between AIP and physical dysfunction, thus deserving further attention.

**Figure 3:**
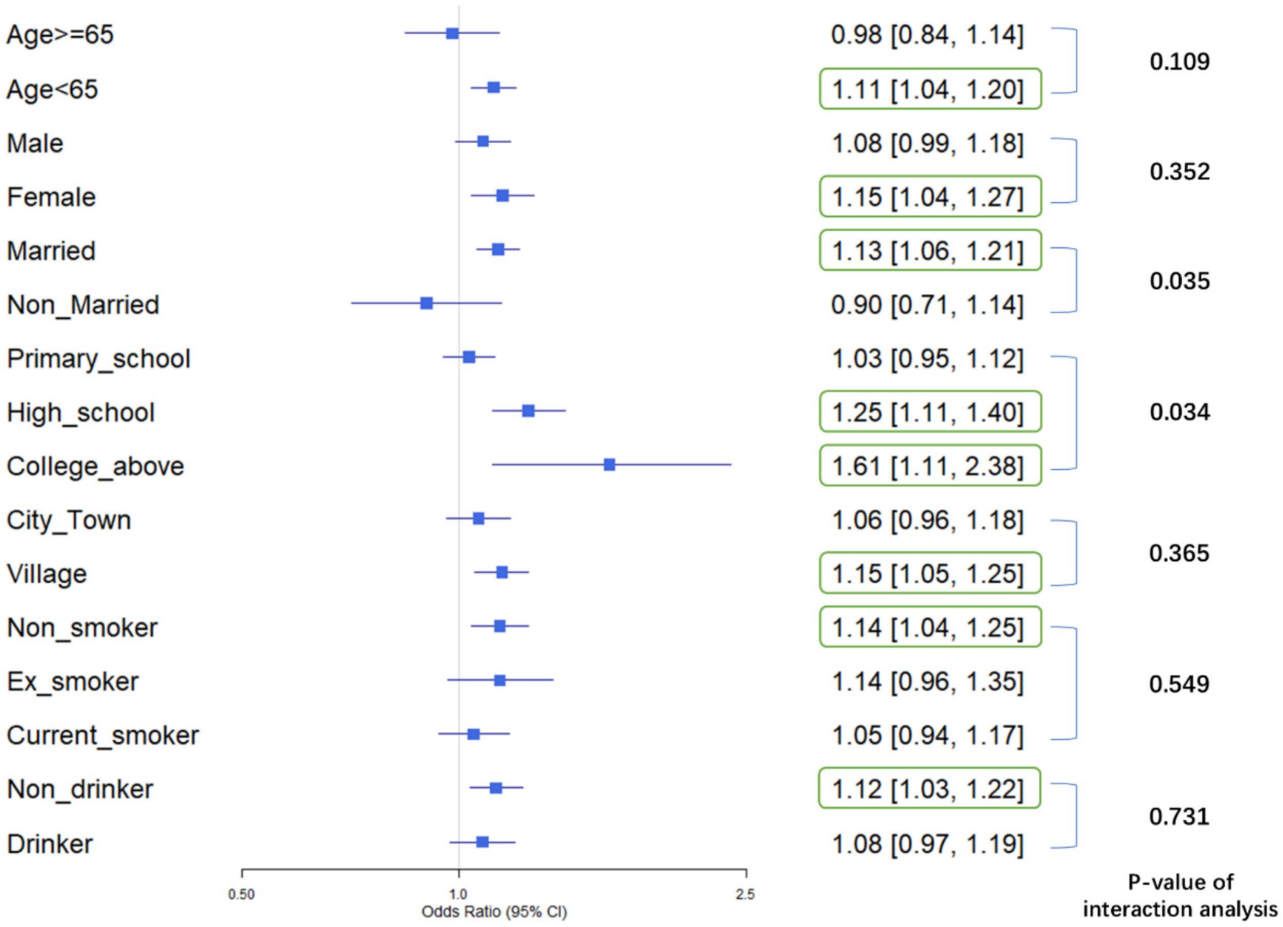
Forest plot of stratified associations and interaction effects: AIP and physical dysfunction across sociodemographic and behavioral subgroups: This plot displays odds ratios (ORs) and 95% confidence intervals (CIs) for physical dysfunction across subgroups defined by age, gender, marital status, education level, residence, smoking, and drinking status. Subgroups with statistically significant associations (*p* < 0.05, CI excluding 1.0) are highlighted: AIP was positively linked to physical dysfunction risk in individuals under 65 years old, females, married adults, those with high school or college/above education, rural residents, non-smokers, and non-drinkers. Marital status (*p* = 0.035) and education level (*p* = 0.034) showed significant interaction effects, indicating they modify the association between AIP and physical dysfunction.

Above all, the stratified analysis showed that AIP was significantly associated with an increased risk of physical dysfunction in subgroups such as individuals under 65 years old, females, married people, those with a high school education, those with a college education or higher, rural residents, non-smokers, and non-drinkers (*p* < 0.05), while no significant association was found in other subgroups. Interaction analysis indicated that marital status (*p* = 0.035) and education level (*p* = 0.034) had significant interaction effects, acting as effect-modifying factors. Their different subgroups significantly altered the association between AIP and physical dysfunction, which deserved attention (Figure 3).

## 4 Discussion

In this study, we analyzed cross-sectional data from 11,369 participants in the CHARLS database to explore, for the first time, the relationship between the Atherogenic Index of Plasma (AIP) and physical dysfunction, while systematically assessing the moderating effects of demographic, health behavior, and social factors. The overall results revealed significant differences in baseline characteristics, health status, and lifestyle behaviors between the physical dysfunction group and the non-dysfunction group. Moreover, AIP was positively associated with physical dysfunction in both continuous and categorical analyses, suggesting AIP may serve as an independent risk factor with potential predictive value.

The baseline data revealed substantial differences between participants with and without physical dysfunction, underscoring the multifactorial nature of functional decline. Although the CHARLS questionnaire used in this study to assess the difficulty of completing 9 daily physical activities is based on respondents’ subjective reports, it has been repeatedly validated in multiple nationally representative studies such as CHARLS and other large-scale international aging cohort studies, demonstrating good reliability and validity (15). While subjective measurement may introduce gender-related, cultural, and psychosocial biases—for instance, females are generally more inclined to report dysfunction—the practical and economic advantages of this subjective assessment approach in large-scale population studies have been widely acknowledged (16, 17), and it is also recognized to have considerable advantages in the macro-assessment of dysfunction among middle-aged and older adult populations. Specifically, baseline characteristics highlighted significant group differences (all *p* < 0.05): the dysfunction group was older, more female, less educated (college+), less married, and more rural-residing, with a higher burden of ≥2 chronic conditions but lower smoking/drinking rates. Metabolically, they exhibited elevated AIP mean, higher proportion in the top AIP quartile, and greater AIP per IQR, with no BMI difference. Collectively, these findings indicate that physical dysfunction is influenced not only by biological aging but also by socioeconomic disparities and lifestyle factors, providing a solid preliminary basis for further regression analyses (18, 19).

Regression analyses consistently demonstrated a significant positive association between AIP and physical dysfunction across all models. In both the unadjusted model and those adjusted for demographic factors (Model 2) and additional covariates such as health behaviors and chronic diseases (Model 3), each interquartile range increase in AIP corresponded to a marked rise in the risk of physical dysfunction (20). Notably, participants in the highest AIP quartile exhibited a significantly greater risk (OR 1.279) compared to those in the lowest quartile. This clear dose–response relationship underscores the cumulative metabolic burden imposed by dyslipidemia (21). As a composite indicator reflecting both triglyceride and HDL-C levels, elevated AIP values signal a detrimental lipid profile—characterized by high triglycerides and low HDL-C—that is known to contribute to atherosclerosis and impaired vascular function (22). Such vascular impairments may lead to reduced blood flow to skeletal muscles, accelerated muscle atrophy, and overall diminished physical performance (23).

Importantly, the persistent association between AIP and physical dysfunction, even after adjusting for key covariates such as chronic conditions, smoking, and alcohol consumption, highlights AIP’s potential role as an independent predictor of functional decline. Beyond its established role in cardiovascular risk, AIP offers valuable insights into systemic metabolic health and the aging process (24). As a simple and cost-effective biomarker, the atherogenic index of plasma (AIP) holds unique promise for clinical screening. Unlike conventional lipid markers such as low-density lipoprotein cholesterol (LDL-C) and total cholesterol (TC), AIP serves as a composite measure of triglycerides (TG) and high-density lipoprotein cholesterol (HDL-C), offering enhanced sensitivity to lipid metabolism disorders and insulin resistance—pathophysiological states strongly linked to functional decline in older adults. Emerging evidence suggests that AIP demonstrates stronger predictive associations than individual lipid indices in certain clinical contexts, such as evaluating cardiovascular risk, insulin resistance, and skeletal muscle loss (25, 26), providing a robust theoretical basis for its clinical utility. Additionally, its accessibility and cost-effectiveness further enhance feasibility in large-scale screening programs. This dual advantage enables the early identification of individuals at risk for functional decline, thereby facilitating timely interventions—ranging from lifestyle modifications to targeted metabolic management—to mitigate long-term disability (27).

Using a restricted cubic spline regression model, we further unveiled a non-linear relationship between the atherogenic index of plasma (AIP) and the risk of physical dysfunction. When AIP values remain below approximately 0.37, the associated risk remains relatively stable (28). However, once this threshold is exceeded, the risk of physical dysfunction rises sharply. This observation aligns with the broader perspective that chronic lipid imbalances gradually lead to functional impairment through multiple interconnected mechanisms. Specifically, the non-linear relationship supports the use of AIP as an early warning biomarker for functional decline (20). Evidence from ROC curve analyses indicates that an optimal AIP cutoff value of approximately 0.37 can be determined—this corresponds to a particular TG/HDL-C ratio (2.34)—and this threshold has been confirmed by numerous studies to be associated with endothelial dysfunction and microvascular damage (29). Overall reference values suggest that a TG/HDL-C ratio above 2.5 to 3.0 should raise some concern, as it may indicate the presence of insulin resistance, metabolic syndrome, or an increased risk of cardiovascular diseases. Some studies have found gender differences in lipid metabolism, suggesting that the warning thresholds may vary; for example, the alert value for men might be above 3.0, while for women it might be above 2.5 (30). Our study’s finding of a TG/HDL-C ratio of 2.34 suggests that physical dysfunction may emerge before overt dyslipidemia becomes apparent, highlighting the need for earlier monitoring and preventive intervention regarding lipid levels (31).

From a mechanistic standpoint, one potential pathway involves microcirculatory disturbances. An elevated AIP reflects a pro-atherogenic lipid profile—characterized by high triglycerides and low HDL-C—that may lead to thickening of the capillary basement membrane and subsequent impairment of skeletal muscle perfusion (32). In fact, some research indicates that for every 0.1-unit increase in AIP, skeletal muscle oxygen uptake may decrease by as much as 5.3%, thereby reducing oxygen delivery and compromising muscle endurance and recovery (33). Another plausible mechanism is mitochondrial dysfunction. Increases in AIP have been associated with a reduction in skeletal muscle mitochondrial DNA copy number, potentially due to disruptions in the AMPK/PGC-1α signaling pathway—a critical regulator of mitochondrial biogenesis and cellular energy homeostasis (34). Such mitochondrial impairment can diminish muscle strength and endurance, accelerating the decline in physical function. Furthermore, systemic inflammation appears to play a crucial role. Elevated AIP levels have been linked to significantly higher levels of interleukin-6 (IL-6), suggesting a state of chronic low-grade inflammation (35). Chronic inflammation is a well-established driver of sarcopenia and muscle wasting, as it disrupts protein metabolism, increases muscle catabolism, and impairs muscle regeneration, all of which contribute to the deterioration of physical function (36). In summary, these findings underscore the importance of early clinical intervention. Identifying and managing individuals with AIP values exceeding 0.37 could be key to mitigating the adverse effects of dyslipidemia on physical function. Early intervention (e.g., lipid modulation, lifestyle modification) at this critical threshold may delay or even prevent further decline in physical function, ultimately reducing the risk of long-term disability.

When analyzed in isolation, AIP exhibited limited discriminative capacity (AUC = 0.525) for physical dysfunction; however, when integrated into a multivariable model adjusted for key covariates—including age, chronic conditions, and socioeconomic factors—the AUC improved to 0.748, demonstrating that AIP contributes meaningfully to predicting dysfunction within a context of relevant demographic and health-related determinants, with moderate to good overall predictive efficacy. Single-marker screening tools often struggle to identify all individuals with physical dysfunction due to inherent trade-offs between sensitivity and specificity. In clinical practice, we propose using an AIP threshold of ≥0.37 as a cautionary marker in senior health assessments. However, ROC analysis revealed a moderate AUC of 0.748 for AIP alone, indicating its predictive performance falls within the “moderate” range. This suggests that relying solely on AIP may lead to either missed cases (due to insufficient sensitivity) or false alarms (due to limited specificity), as single biomarkers inherently balance these two critical diagnostic metrics. To address this limitation, integrating AIP with complementary clinical indicators—such as age (reflecting age-related physiological decline), chronic disease status (indicating organ dysfunction burden), and inflammatory markers (signaling systemic stress)—is essential. Each of these factors captures distinct pathophysiological pathways: age reflects the cumulative effects of biological aging, chronic diseases directly impact functional reserve, and inflammation underscores systemic stress responses. By incorporating AIP into a comprehensive risk model that includes demographic factors (e.g., age, education), health status (chronic diseases, lifestyle behaviors like physical activity and smoking), and socioeconomic determinants, the predictive accuracy and clinical utility of the screening tool can be significantly enhanced. Such a multifactorial approach better aligns with the complex, multisystem nature of physical dysfunction, minimizing diagnostic errors and improving risk stratification in real-world clinical settings (23, 37, 38).

The stratified analysis revealed distinct subgroup vulnerabilities in the association between AIP and physical dysfunction, offering nuanced insights into how sociodemographic and behavioral factors modulate this relationship. Middle-aged adults (<65 years) exhibited a pronounced positive association, suggesting that midlife exposure to AIP-related lipid dysregulation may precipitate early functional decline in the absence of adaptive aging mechanisms (39) or amid reduced resilience to subclinical deficits (40), whereas in older adults, competing risks like multimorbidity or frailty may overshadow AIP’s unique impact. Females showed a significant sex-specific effect, potentially rooted in biological sensitivities [e.g., estrogen-mediated lipid metabolism (41) and vascular responsiveness to oxidative stress (42)] or sociocultural factors (e.g., caregiving roles or sedentary lifestyles increasing the physical demands placed on their bodies under metabolic compromise). Married individuals, despite potential social support benefits, displayed stronger associations, a paradox likely driven by shared lifestyle risks (e.g., homogeneous diets, sedentary cohabitation) or caregiving stress accelerating the decline in physical function among those with elevated AIP. For nonsmokers and non-drinkers, AIP emerged as a clearer marker of lipid metabolism–driven dysfunction, unobscured by tobacco/alcohol-related pathologies like inflammation or hepatic injury, while in smokers/drinkers, competing harmful biological pathways [e.g., inflammation, hepatic injury (43, 44)] likely overshadowed AIP’s role. Rural residents, who often have limited access to healthcare or are exposed to environmental stressors (e.g., intensive physical labor, air pollution), may exhibit a stronger association between AIP and physical dysfunction; however, this hypothesis requires validation with context-specific lifestyle and environmental data.

Notably, education level (*p* = 0.034) and marital status (*p* = 0.035) exhibited significant interaction effects, acting as key effect modifiers that altered the strength of the AIP-dysfunction association across their subgroups. The modifying effect of education level was evident as the impact of AIP on physical dysfunction strengthened with higher educational attainment, particularly among those with high school or college-level education, likely linked to differences in health behaviors, occupational environments, and resource access: higher-educated individuals often engage in sedentary, high-stress cognitive labor with limited physical activity and greater sensitivity to functional decline due to higher health awareness, while lower-educated individuals’ habitual physical labor may buffer AIP-related metabolic risks and mask direct effects on physical function. As a marker of socioeconomic status, education reflects lifelong environmental stressors (e.g., workplace competition) and health maintenance capabilities, amplifying or attenuating AIP’s biological effects. Marital status exhibited a counterintuitive role, with a stronger positive association in married individuals, possibly driven by shared unhealthy lifestyles exacerbating lipid dysregulation and caregiving responsibilities increasing physical/emotional burdens; while marriage is associated with social support, its protective effects may be offset by shared risks or caregiving stress, requiring contextual evaluation of spouse health and family roles, whereas unmarried individuals’ independent lifestyles reduce amplifying factors for this association. These findings highlight that AIP’s clinical value as a metabolic marker must be interpreted within social contexts: elevated AIP in highly educated married individuals signals urgent intervention due to overlapping biological (lipid dysregulation) and social (sedentary work, caregiving) risks, while lower-educated unmarried populations require focus on determinants like physical activity; future research should explore pathways (e.g., inflammation, neuroendocrine mechanisms) through which education and marriage influence AIP’s effects to develop integrated “social-biological” strategies such as couple-based health programs or workplace initiatives for educated groups to address subgroup-specific vulnerabilities.

While this study significantly advances our understanding of AIP’s role in functional decline, several limitations must be acknowledged. First, as a cross-sectional design, it cannot establish causality or temporal sequence, the directionality of the AIP-physical dysfunction association (e.g., whether elevated AIP precedes functional decline or vice versa) remains unclear. Prospective cohort studies, including Mendelian randomization designs, are essential to determine the causal pathway. Second, the relatively small sample size in the high-AIP subgroup resulted in wider confidence intervals (CIs), possibly related to the heterogeneity of the high AIP population (e.g., combined diabetes, obesity), which potentially reduce the precision of risk estimates in this range. Future longitudinal studies with larger samples are needed to validate the causal sequence between elevated AIP and physical dysfunction, addressing the cross-sectional design’s limitation in inferring temporal relationships. Third, unmeasured confounders—such as detailed dietary intake, objectively measured physical activity, sedentary behavior and differences between individuals in health insurance coverage and access to healthcare services—could have influenced the observed associations. Fourth, a notable limitation is the absence of subgroup analyses by distinct functional domains (e.g., mobility, self-care, instrumental activities), which precluded clarification on whether the observed gender disparities in physical dysfunction stem from specific impairments. While the higher proportion of females reporting dysfunction in Table 1 may reflect biological differences [e.g., decreased estrogen levels accelerate bone mineral density loss (45)], sociocultural factors (e.g., caregiving roles, health perception norms), or reporting biases [e.g., women may be more sensitive to changes in physical functioning (46)], our composite outcome and cross-sectional design could not disentangle these pathways. The self-reported assessment of daily activities (e.g., gait, stair climbing, chair rise), aligned with validated tools in CHARLS and other epidemiological studies, efficiently captures population-level functional status but is subject to gender-sensitive subjectivity. Future studies using objective functional measures—such as the Short Physical Performance Battery (SPPB) (47, 48), grip strength (49, 50) or 400-meter walk test (51, 52)—are warranted to validate these associations and pinpoint the dimensional origins of sex-specific vulnerability. Incorporating both subjective self-reports and objective assessments would enhance understanding of how AIP relates to distinct aspects of functional decline, informing targeted interventions for high-risk subgroups. An elevated AIP reflects a dysregulated lipid profile associated with lipid deposition, inflammatory responses, endothelial dysfunction, and atherosclerosis (53). These processes can lead to microcirculatory disturbances and reduced muscle perfusion, ultimately contributing to physical decline. Furthermore, the reliance on self-reported measures of physical dysfunction, although widely validated in large cohort studies, may still introduce measurement bias. Future research should focus on developing dynamic, multidimensional risk prediction models that incorporate AIP alongside additional biomarkers and real-time data from wearable devices, thereby enhancing early detection and monitoring. Investigations into the effects of novel lipid-lowering therapies, such as Proprotein convertase subtilisin/kexin type 9(PCSK9) inhibitors, on AIP-associated functional decline are also warranted. Establishing AIP-based stratified management pathways at the community level could yield significant public health benefits through targeted interventions. Ultimately, integrating AIP into routine midlife health assessments may empower policymakers to leverage its cost-effectiveness in promoting healthy aging, thus strengthening the foundation for personalized public health initiatives and interventions.

## 5 Conclusion

This study demonstrated a significant association between the atherogenic index of plasma (AIP) and physical dysfunction, with the risk notably higher among individuals with elevated AIP levels. Stratified analyses showed significant associations between AIP and physical dysfunction in subgroups including adults <65 years, females, married individuals, those with high school/college education, rural residents, non-smokers, and non-drinkers. Marital status and education level significantly modified these associations, acting as effect modifiers. These findings highlight subgroup-specific vulnerabilities and the need for targeted strategies to address AIP-related functional health risks. As a simple and accessible biomarker, AIP shows promise for use in older adults screening and risk assessment for functional decline in aging populations. Future research should further investigate the interaction between AIP and other health indicators, as well as the underlying biological mechanisms, to strengthen the evidence base for early intervention and the development of personalized healthcare strategies.

## Data Availability

The original contributions presented in the study are included in the article/Supplementary material, further inquiries can be directed to the corresponding author.

## Footnotes

^1^ http://www.stats.gov.cn/english/

^2^ http://charls.pku.edu.cn/
