# Supplementary figures and images for: Association between atherogenic index of plasma and physical dysfunction: a cross-sectional study of middle-aged and older adults in China
Source: Front Public Health. 2025 May 30;13:1580340. doi: 10.3389/fpubh.2025.1580340 (PMC12162623; doi:10.3389/fpubh.2025.1580340)

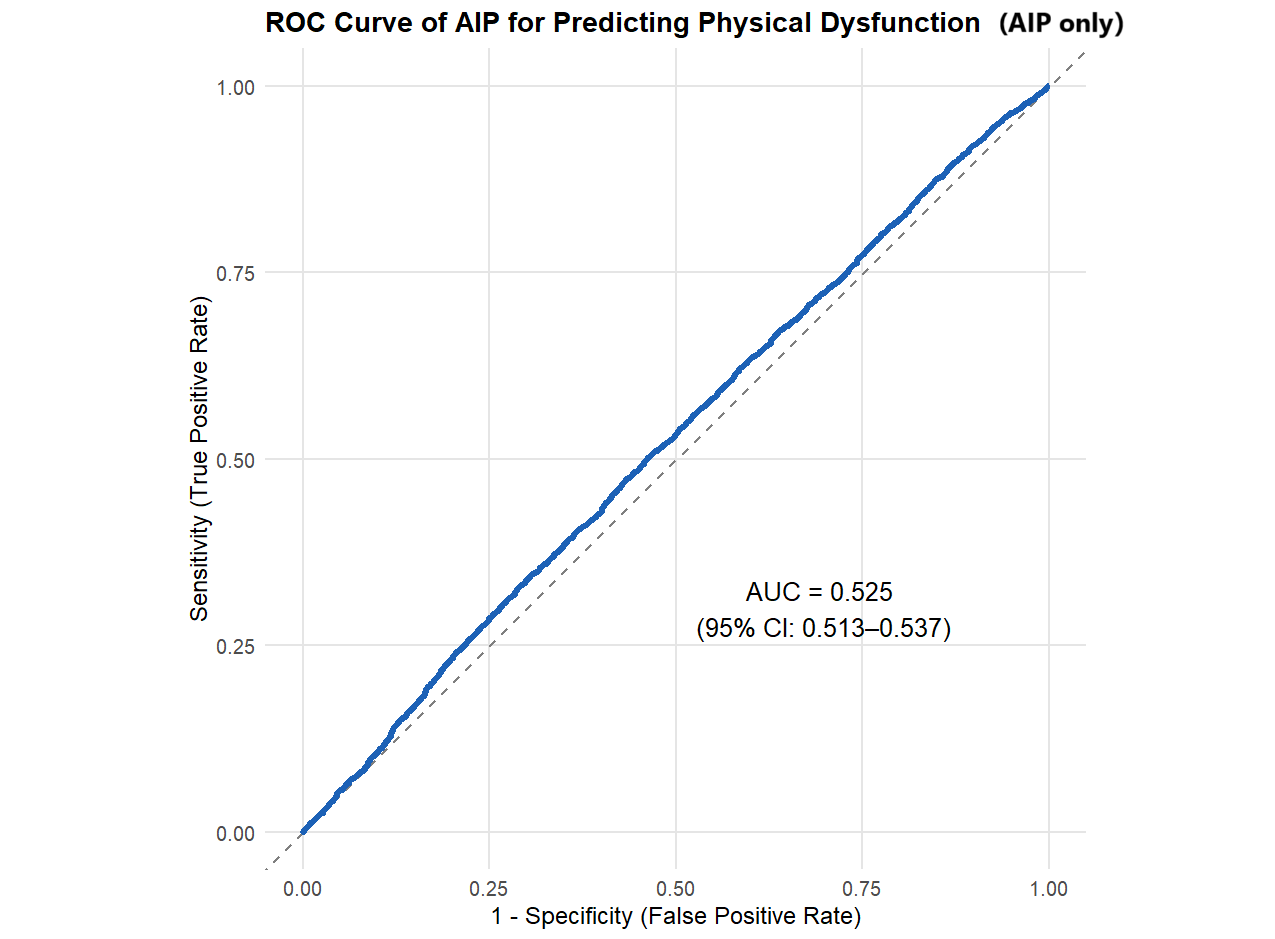

Supplement: Supplementary file 1 [file Image_1.png]
